# Antiparasitic activity of furanyl *N*-acylhydrazone derivatives against *Trichomonas vaginalis*: *in vitro* and *in silico* analyses

**DOI:** 10.1186/s13071-020-3923-8

**Published:** 2020-02-11

**Authors:** Mirna Samara Dié Alves, Raquel Nascimento das Neves, Ângela Sena-Lopes, Micaela Domingues, Angela Maria Casaril, Natália Vieira Segatto, Thaís Cristina Mendonça Nogueira, Marcus Vinicius Nora de Souza, Lucielli Savegnago, Fabiana Kömmling Seixas, Tiago Collares, Sibele Borsuk

**Affiliations:** 1 Laboratório de Biotecnologia Infecto-parasitária, Centro de Desenvolvimento Tecnológico, Biotecnologia, UFPel, Pelotas, RS 96010-900 Brazil; 2 Laboratório de Neurobiotecnologia, Centro de Desenvolvimento Tecnológico, Biotecnologia, UFPel, Pelotas, RS 96010-900 Brazil; 3 Laboratório de Biotecnologia do Câncer, Centro de Desenvolvimento Tecnológico, Biotecnologia, UFPel, Pelotas, RS 96010-900 Brazil; 4 Instituto de Tecnologia em Fármacos-Far-Manguinhos, Fiocruz-Fundação Oswaldo Cruz, Rio de Janeiro, RJ 21041-250 Brazil; 5 Programa de Pós-Graduação em Química, Instituto de Química, Universidade Federal do Rio de Janeiro, Cidade Universitária, Rio de Janeiro, RJ 21945-970 Brazil

**Keywords:** Antiparasitic, Lipid peroxidation, Molecular docking, Trichomoniasis, Trichomonacidal

## Abstract

**Background:**

*Trichomonas vaginalis* is the causative agent of trichomoniasis, which is one of the most common sexually transmitted diseases worldwide. Trichomoniasis has a high incidence and prevalence and is associated with serious complications such as HIV transmission and acquisition, pelvic inflammatory disease and preterm birth. Although trichomoniasis is treated with oral metronidazole (MTZ), the number of strains resistant to this drug is increasing (2.5–9.6%), leading to treatment failure. Therefore, there is an urgent need to find alternative drugs to combat this disease.

**Methods:**

Herein, we report the *in vitro* and *in silico* analysis of 12 furanyl *N*-acylhydrazone derivatives (PFUR 4, a-k) against *Trichomonas vaginalis*. *Trichomonas vaginalis* ATCC 30236 isolate was treated with seven concentrations of these compounds to determine the minimum inhibitory concentration (MIC) and 50% inhibitory concentration (IC_50_). In addition, compounds that displayed anti-*T. vaginalis* activity were analyzed using thiobarbituric acid reactive substances (TBARS) assay and molecular docking. Cytotoxicity analysis was also performed in CHO-K1 cells.

**Results:**

The compounds PFUR 4a and 4b, at 6.25 µM, induced complete parasite death after 24 h of exposure with IC_50_ of 1.69 µM and 1.98 µM, respectively. The results showed that lipid peroxidation is not involved in parasite death. Molecular docking studies predicted strong interactions of PFUR 4a and 4b with *T. vaginalis* enzymes, purine nucleoside phosphorylase, and lactate dehydrogenase, while only PFUR 4b interacted *in silico* with thioredoxin reductase and methionine gamma-lyase. PFUR 4a and 4b led to a growth inhibition (< 20%) in CHO-K1 cells that was comparable to the drug of choice, with a promising selectivity index (> 7.4).

**Conclusions:**

Our results showed that PFUR 4a and 4b are promising molecules that can be used for the development of new trichomonacidal agents for *T. vaginalis*.
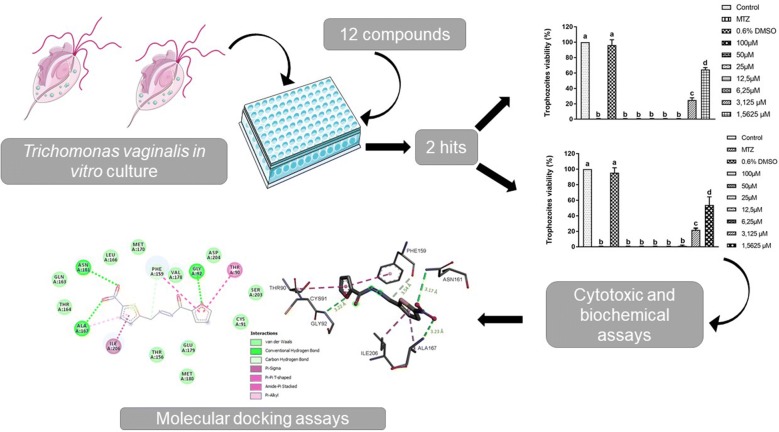

## Background

*Trichomonas vaginalis* is an anaerobic, flagellated protozoan parasite, which infects the urogenital tract of humans causing trichomoniasis, the most prevalent non-viral sexually transmitted infection worldwide [1]. Trichomoniasis has an estimated incidence rate of 276 million cases per year [1]. In Brazil, the prevalence varies between 2.5% and 20% in women [1–6]. *Trichomonas vaginalis* has a greater impact on public health as it is associated with increased risk of preterm delivery, pelvic inflammatory disease [7] and HIV acquisition and transmission [8, 9]. It is important to note that non-adherence and/or noncompliance to treatment for trichomoniasis, which happens frequently, helps in maintaining the chain of infection [8].

For more than five decades, in most countries, the treatment for *T. vaginalis* has been restricted to 5-nitroimidazoles, with metronidazole (MTZ) and tinidazole (TNZ) being the only drugs approved by the U.S. Food and Drug Administration Agency [9, 10]. The activity of MTZ depends on the reduction of its nitro group by hydrogenosome metabolism and by flavin enzyme thioredoxin reductase (TrxR) [11]; however, the increasing number of *T. vaginalis* isolates resistant to 5-nitroimidazoles is a major concern [12–14] representing 2.5–9.6% of clinical cases [12, 13, 15]. Nonetheless, inhibition of TrxR and other enzymes such as lactate dehydrogenase (LDH), methionine gamma-lyase (MGL), and purine nucleoside phosphorylase (PNP) also seem to be a rational approach for chemotherapy against *T. vaginalis* due to their importance on protozoan survival [16–18].

In this context, there is a need to identify new molecules with potential trichomonacidal activity as an alternative to 5-nitroimidazoles. *N*-acylhydrazone derivatives have been found to have several biological properties, such as antimalarial [19], antitumor [20], antifungal [21] and antibacterial [22] activities, exhibiting good pharmacological potential. In addition, furan and related derivatives represent an important class of heterocyclic compounds that have been reported to exhibit a wide range of biological activities, such as antibacterial, antiviral, anti-inflammatory, antifungal, antitumor, etc. [23–25].

Considering the need for new trichomonacidal compounds, a series of furanyl *N*-acylhydrazone derivatives (PFUR) was designed by molecular hybridization technique combining both pharmacophoric groups, i.e. the *N-*acylhydrazone and furan moieties in the molecules. Therefore, this study was aimed at e the *in vitro* activity and selectivity of 12 synthesized PFUR derivatives against *T. vaginalis* and at identifying their likely binding to *T. vaginalis* proteins *in silico*.

## Methods

### Chemicals

**Fig. 1:**
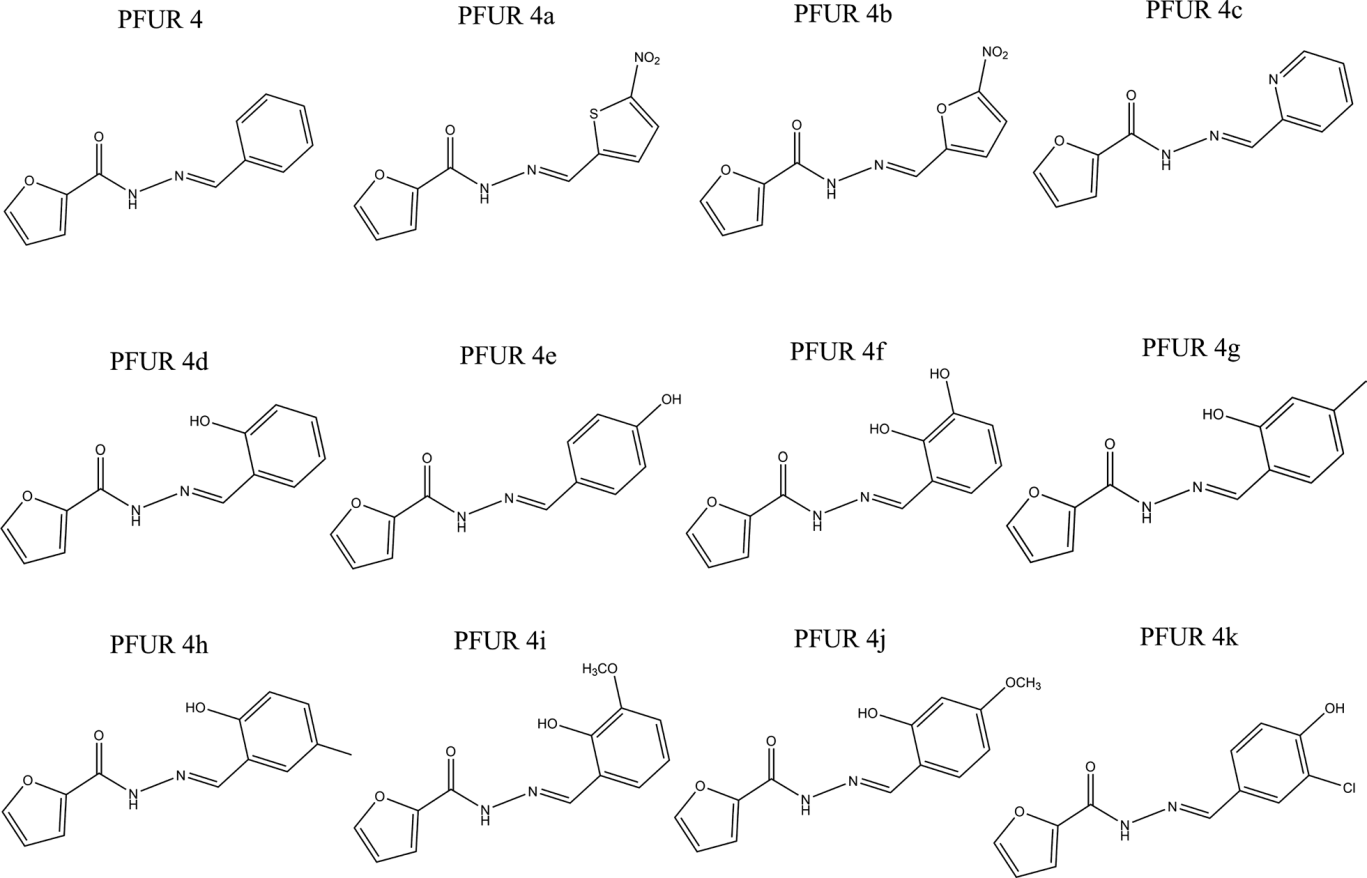
Chemical structures of PFUR 4 and 4a-k synthesized by the reaction of 2-(H_2_NNHCO-furan) and aryl aldehydes (EtOH, RCHO, r.t., 1–72 h, 40–97%)

The synthesis of furanyl *N*-acylhydrazone derivatives was carried out as described by Cardoso et al. [26] and the synthesized samples were kindly provided by Dr Marcus Vinicius Nora de Souza from the Instituto de Tecnologia em Fármacos-Far-Manguinhos, Fiocruz, Brazil. Briefly, the synthesis was carried out by the reaction between 2-(H_2_NNHCO-furan), which were generated from methyl furan-2-carboxylate, and arylaldehydes resulting in the formation of 12 derivatives, which are as follows: PFUR 4 (precursor), PFUR 4a, PFUR 4b, PFUR 4c, PFUR 4d, PFUR 4e, PFUR 4f, PFUR 4g, PFUR 4h, PFUR 4i, PFUR 4j and PFUR 4k (Fig. 1).

### *Trichomonas vaginalis* culture

The isolate *T. vaginalis* Donne ATCC 30236 that is susceptible to MTZ was axenically grown in pre-warmed trypticase-yeast extract-maltose (TYM) medium without agar (pH 6.0), supplemented with 10% sterile bovine serum (SBS) (previously inactivated at 56 °C for 30 min) with 3% streptomycin (5 mg/ml) and incubated at 37 °C under microaerobic conditions [27]. The motility and morphology of the culture were analyzed under light microscopy at 400× magnification. A trypan blue (0.4%) exclusion assay was performed to ensure that minimum viability of 95% and logarithmic growth phase had been achieved before proceeding to antiparasitic and biochemical assays.

### Anti-*T. vaginalis* assay

Drug susceptibility assays were performed to analyze the antiparasitic potential of 12 PFUR compounds against *T. vaginalis* as previously described [28, 29]. All compounds were diluted in dimethylsulfoxide (DMSO). For the assay, 96-well microtiter plates were seeded with 2.6 × 10^5^ trophozoites/ml of TYM (initial density), then PFUR drugs were added into the wells at a concentration of 100 µM, and the plates were incubated at 37 °C with 5% CO_2_ for 24 h. Following incubation, an aliquot of trophozoites and trypan blue (0.4%) (1:1, v/v) was counted in the Neubauer chamber to determine trophozoite motility, morphology and viability through trypan blue dye exclusion. Three controls were used in each assay: a negative control containing only trophozoites; a 0.6% DMSO control (vehicle for solubilization); and a positive control containing MTZ at 100 µM (Sigma-Aldrich, St. Louis, Missouri, USA). Only the compounds that reduced the viability of parasites by 100% were used in the subsequent experiments.

The minimum inhibitory concentration (MIC) and 50% inhibitory concentration (IC_50_) values against *T. vaginalis* were established in the same conditions described previously with trophozoites exposed for 24 h to PFUR concentrations ranging from 1.5625 µM to 100 µM. The MIC was confirmed by transferring the culture solutions contained in 96-well plates from MIC and from concentrations directly below and above, as well as controls, to tubes containing 1.5 ml of fresh TYM medium, with SBS and antibiotics. Tubes were then re-incubated at 37 °C with 5% CO_2_ for 96 h and trophozoites were counted in the Neubauer chamber every 24 h to confirm MIC. The IC_50_ was calculated using GraphPad Prism 7.0 software.

A kinetic growth curve was established to understand the time required for the antiparasitic activity of compounds on *T. vaginalis* cultures. 96-well plates were prepared as previously described, compounds were added at MIC, and the plates were incubated at 37 °C with 5% CO_2_ for 96 h. The viability of trophozoites was observed under light microscopy and the growth analysis was performed at 1, 6, 12, 24, 48, 72 and 96 h using trypan blue (0.4%) dye exclusion and motility and morphology analysis. All assays were performed in three independent set-ups in triplicate and the results were expressed as a percentage of viable trophozoites compared with the negative control.

### Thiobarbituric acid reactive substances (TBARS) assay

The treatment and control groups were evaluated for the formation of TBARS in an acid-heating reaction, and malondialdehyde (MDA) was chosen as the biomarker for lipid peroxidation. The assay was performed as described by Ohkawa et al. [30] with modifications. For this, 96-well plates were seeded with *T. vaginalis* (2.6 × 10^5^ trophozoites/ml), the compounds (at MIC) and controls were added, and plates were incubated at 37 °C with 5% CO_2_ for 24 h. After incubation, aliquots of *T. vaginalis* control and treatment groups were incubated with 0.8% TBA, acetic acid/HCl (pH 3.4), and 8.1% SDS (sodium dodecyl sulfate) at 95 °C for 2 h. The absorbance of the samples was measured at 532 nm and the results were expressed as nmol MDA/10^5^ trophozoites.

### Molecular docking

Molecular docking of PFUR compounds was performed using crystal structure of *T. vaginalis* purine nucleoside phosphorylase (TvPNP; PDB 1Z36), lactate dehydrogenase (TvLDH; PDB 5A1T), and methionine gamma-lyase (TvMGL; PDB 1E5E), which were retrieved from RSCB protein data bank (http://www.rcsb.org/). The structure of *T. vaginalis* thioredoxin reductase (TvTrxR) was constructed using the server SWISS-MODEL [31] based on the UniProt entry A2DSU2. Target protein structures were prepared by removing ligands, ions, and water with CHIMERA 1.5.3 [32] software. Non-polar hydrogen atoms were merged, Gasteiger atomic charges were assigned, the atom type of protein structures was specified, and the molecular docking simulation was performed using Autodock Vina 1.1.2 software [33]. The binding sites were defined by the co-complexed ligands in the crystal structure and the grid box was set up according to the corresponding residues. PFUR 4a, PFUR 4b, and MTZ were built and optimized in the software Avogadro 1.1.1 [34]. Followed by structural optimization, the ligands were prepared for docking by merging non-polar hydrogen atoms, defining rotatable bonds and assigning partial atomic charge using AutoDock Tools version 1.5.6 [35]. Docking poses of the investigated compounds were visualized using Accelrys Discovery Studio 3.5.

### Cell culture

Chinese Hamster Ovary (CHO-K1) cells obtained from the Rio de Janeiro Cell Bank (PABCAM, Federal University of Rio de Janeiro, RJ, Brazil) were grown as a monolayer in Dulbecco’s Modified Eagle’s Medium (DMEM) (Vitrocell Embriolife, São Paulo, Brazil), supplemented with 10% fetal bovine serum (Vitrocell Embriolife), 1% l-glutamine, and 1% penicillin/streptomycin at 37 °C in an atmosphere of 95% humidified air and 5% CO_2_.

### Cytotoxicity assay

The cytotoxicity assay was performed following a method previously described [28, 36]. CHO-K1 cell line viability and proliferation were determined by measuring the formation of water-insoluble formazan as a result of 3-(4,5-dimethythiazol-2-yl)–2,5-diphenyl tetrazolium bromide (MTT) reduction. In 96-well plates, 2 × 10^4^ cells were seeded per well and grown at 37 °C, 5% CO_2_ for 24 h prior to the cell viability assay. After that, the cells were treated with compounds at 1.5625, 3.125, 6.25 and 12.5 μM for another 24 h at 37 °C, 5% CO_2_. In addition, 0.6% DMSO and MTZ at 100 μM were added as controls. After incubation, MTT (Sigma-Aldrich) at 5 mg/ml was added per well and the plates were re-incubated for 3 h at 37 °C, 5% CO_2_. Subsequently, DMSO was added to solubilize formazan crystals. The absorbance was measured on a microplate reader at a wavelength of 492 nm. The inhibition of cell growth was determined according to the formula: Cell proliferation inhibition (%) = (1 − Abs_492 treated cells_/Abs_492 control cells_) × 100. The 50% cytotoxic concentration (CC_50_) was calculated using GraphPad Prism 7.0 software. The assay was performed at least three independent times in triplicate. The selectivity index (SI) was calculated through the ratio CC_50_/IC_50_.

### Statistical analysis

Statistical analysis was carried out by two-way analysis of variance (ANOVA) for the kinetic growth curve and for all other assays by one-way ANOVA, both followed by Tukey’s *post-hoc* test for multiple comparisons between groups. All tests were performed using GraphPad Prism 7.03 software, considering a probability value of *P* < 0.05. Data are expressed as the mean ± standard deviation (SD).

## Results

### Anti-*T. vaginalis* assay

**Fig. 2:**
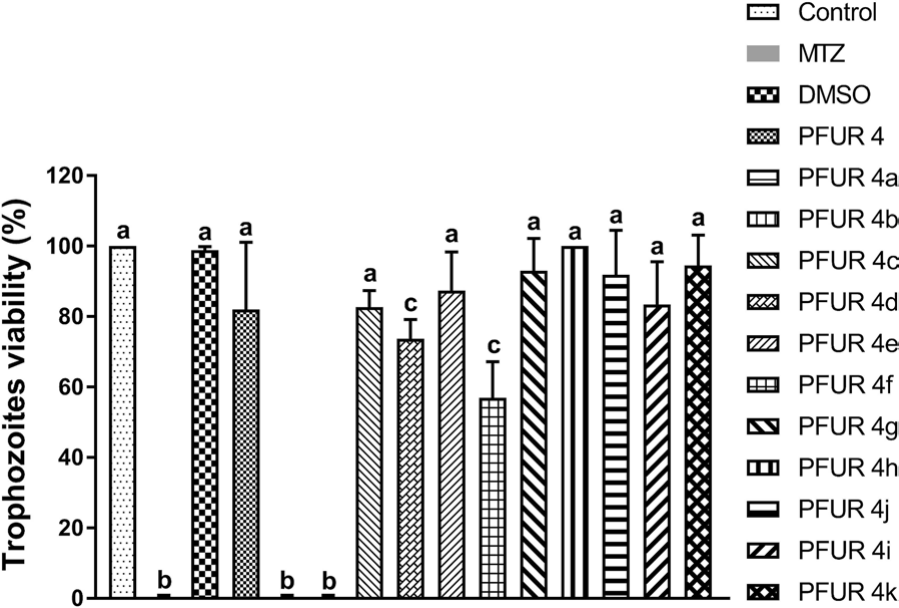
*In vitro* trichomonacidal activity of PFUR 4 and 4a-k at 100 µM against *Trichomonas vaginalis* ATCC 30236 isolate, confirmed by the trypan blue (0.4%) assay after 24 h of exposure: Control (untreated trophozoites), 0.6% DMSO (vehicle for solubilization), MTZ (metronidazole at 100 µM). Viability of 100% in control corresponds to 2.6 × 10^5^ trophozoites/ml. Data are presented as the mean ± standard deviation of at least three independent experiments. Different letters show a significant difference at *P *< 0.05

The screening for trichomonacidal activity showed the potential of two compounds from the PFUR group, 4a and 4b, as both reduced the trophozoites viability by 100% at 100 µM after 24 h (ANOVA: *F*_(14, 30)_ = 57.32, *P* < 0.0001). Moreover, PFUR 4h had no effect on trophozoites growth, PFUR 4d and 4f were able to reduce growth by 26.3% and 43.1%, respectively; however, such reduction was not significant when compared with MTZ, as for other compounds (PFUR 4, 4c, 4e, 4g, 4i-k) viability reduction rates varied from 5.5% to 18% and were not statistically different from the negative control group (ANOVA: *F*_(14, 30) _= 57.32, *P *< 0.0001) (Fig. 2). Unsurprisingly, 0.6% DMSO and negative controls induced no reduction in viability, exhibited negative trypan blue (0.4%) staining, and showed good motility and morphology, whereas treatment with MTZ completely diminished viability and exhibited positive trypan blue staining and negative motility.

**Fig. 3:**
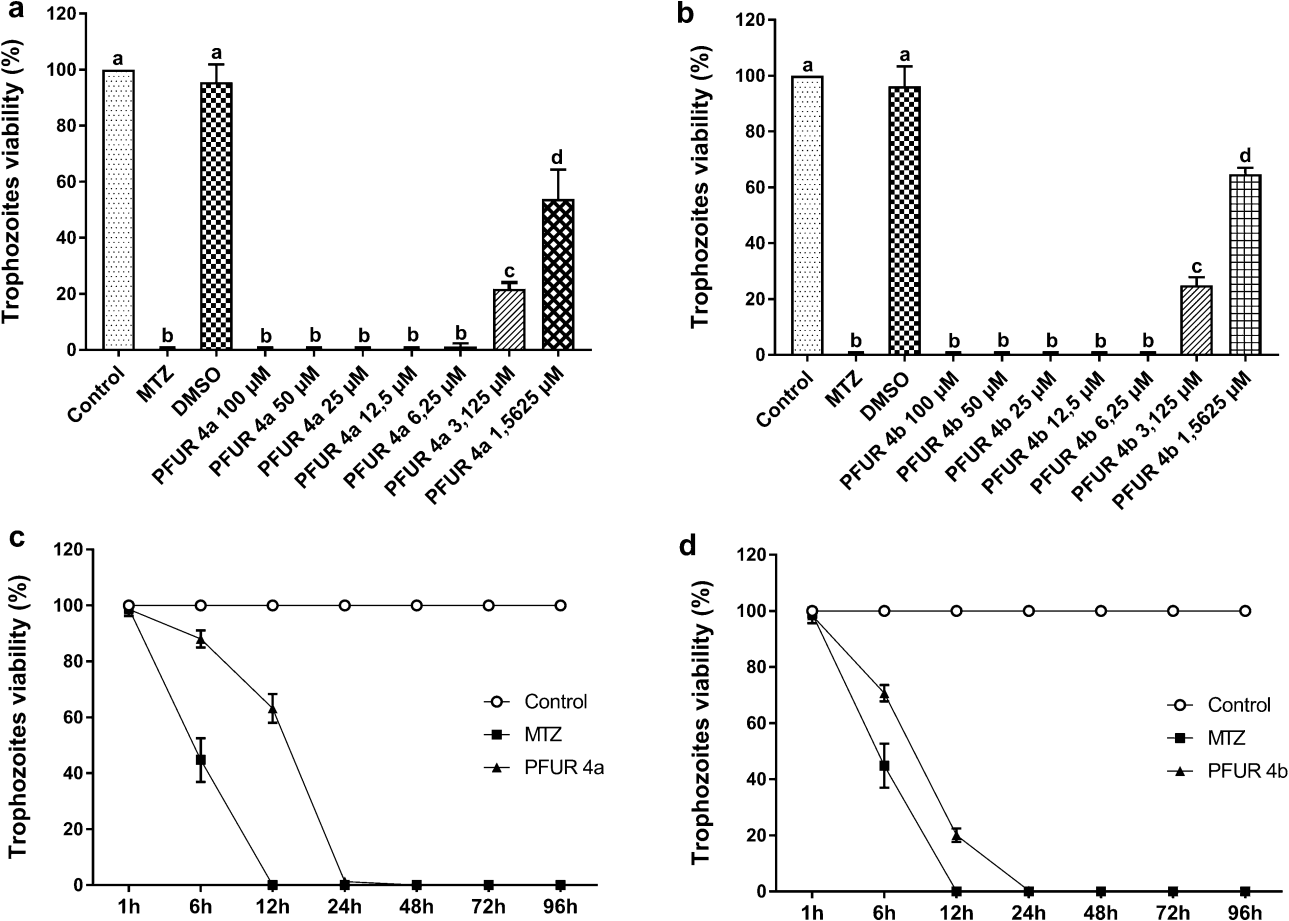
Anti-*Trichomonas vaginalis* assay. MIC and IC_50_ for the antiparasitic activity of PFUR 4a (**a**) and PFUR 4b (**b**) against *Trichomonas vaginalis* ATCC 30236 after exposure to 1.5625, 3.125, 6.25, 12.5, 25, 50 and 100 µM concentrations for 24 h. Kinetic growth curves of *Trichomonas vaginalis* ATCC 30236 after 1, 6, 12, 24, 48, 72 and 96 h of treatment with PFUR 4a (**c**) and PFUR 4b (**d**) at 6.25 µM. Growth was completely inhibited after 24 h. Control (untreated trophozoites), 0.6% DMSO (vehicle for solubilization), MTZ (metronidazole at 100 µM). Viability of 100% in control corresponds to 2.6 × 10^5^ trophozoites/ml. Data are presented as the mean ± standard deviation of at least three independent experiments. Different letters show a significant difference at *P *< 0.05

Considering the full viability reduction caused by PFUR 4a and 4b, these compounds were chosen for antiparasitic and biochemical tests and molecular docking. The tests showed that PFUR 4a (ANOVA: *F*_(9, 20) _= 317.1, *P *< 0.0001) and PFUR 4b (ANOVA: *F*_(9, 20) _= 799.5, *P *< 0.0001) were able to completely reduce trophozoites viability at 6.25 µM (Fig. 3a, b), which was determined as MIC. IC_50_ was statistically found to be 1.69 µM and 1.98 µM for PFUR 4a and PFUR 4b, respectively, after 24 h exposure (Table 1).

**Table 1:** Anti-*Trichomonas vaginalis* activity and cytoxicity effects of furanyl N-acylhydrazone (PFUR) derivatives 4a and 4b

Compound	*Trichomonas vaginalis*		CHO-K1 cells	
	MIC	IC_50_ ± SE^a^	CC_50_	SI
PFUR 4a	6.25	1.69 ± 0.208	> 12.5	> 7.4
PFUR 4b	6.25	1.98 ± 0.121	> 2.5	> 6.3

^a^Standard error (SE) as calculated by GraphPad Prism 7.03 software

*Notes*: All results are expressed in μM, except for SI values. Trophozoites and cells were exposed to PFUR 4a and 4b for 24h for all *in vitro* assays

The data obtained from kinetic growth curve showed that PFUR 4a at 6.25 µM was able to reduce trophozoite growth by 36.8% at 12 h and 98.7% at 24 h; conversely, the negative control maintained full growth at all exposure times and MTZ completely inhibited growth at 12 h exposure (Two-way ANOVA: *F*_(12, 36) _= 299.2, *P* < 0.0001) (Fig. 3c). As for PFUR 4b at 6.25 µM, growth reduction was 79.9% at 12 h and 99.7% at 24 h, whereas for the negative control and MTZ, the profile was the same as described above (Two-way ANOVA: *F*_(12, 36) _= 401.1, *P *< 0.0001) (Fig. 3d). For both compounds, there were no viable trophozoites after 24 h.

### TBARS assay

**Fig. 4:**
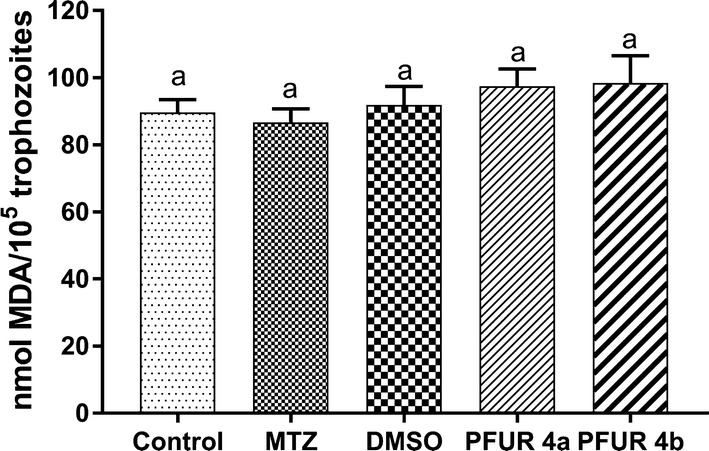
Lipid peroxidation levels measured through thiobarbituric acid reactive substances assay with malondialdehyde (MDA) as a biomarker, after 24 h of exposure to PFUR 4a and 4b, both at 6.25 µM. Control (untreated trophozoites), DMSO (vehicle for solubilization), MTZ (metronidazole at 100 µM). Data are presented as the mean ± standard deviation of at least three independent experiments

Data analysis demonstrated that treatment with 0.6% DMSO did not increase lipid peroxidation levels when compared with the control group (untreated trophozoites). The results also showed that MTZ, PFUR 4a, and PFUR 4b treatments were unable to significantly induce lipid peroxidation levels than those of the control (ANOVA: *F*_(4, 25) _= 4.901, *P*= 0.0047) (Fig. 4).

### Molecular docking

Next, a molecular docking study was carried out with the two most active compounds, PFUR 4a and 4b, to investigate their possible mechanism of action through their *in silico* predicted interaction with proteins that play a role in *T. vaginalis* survival.

**Fig. 5:**
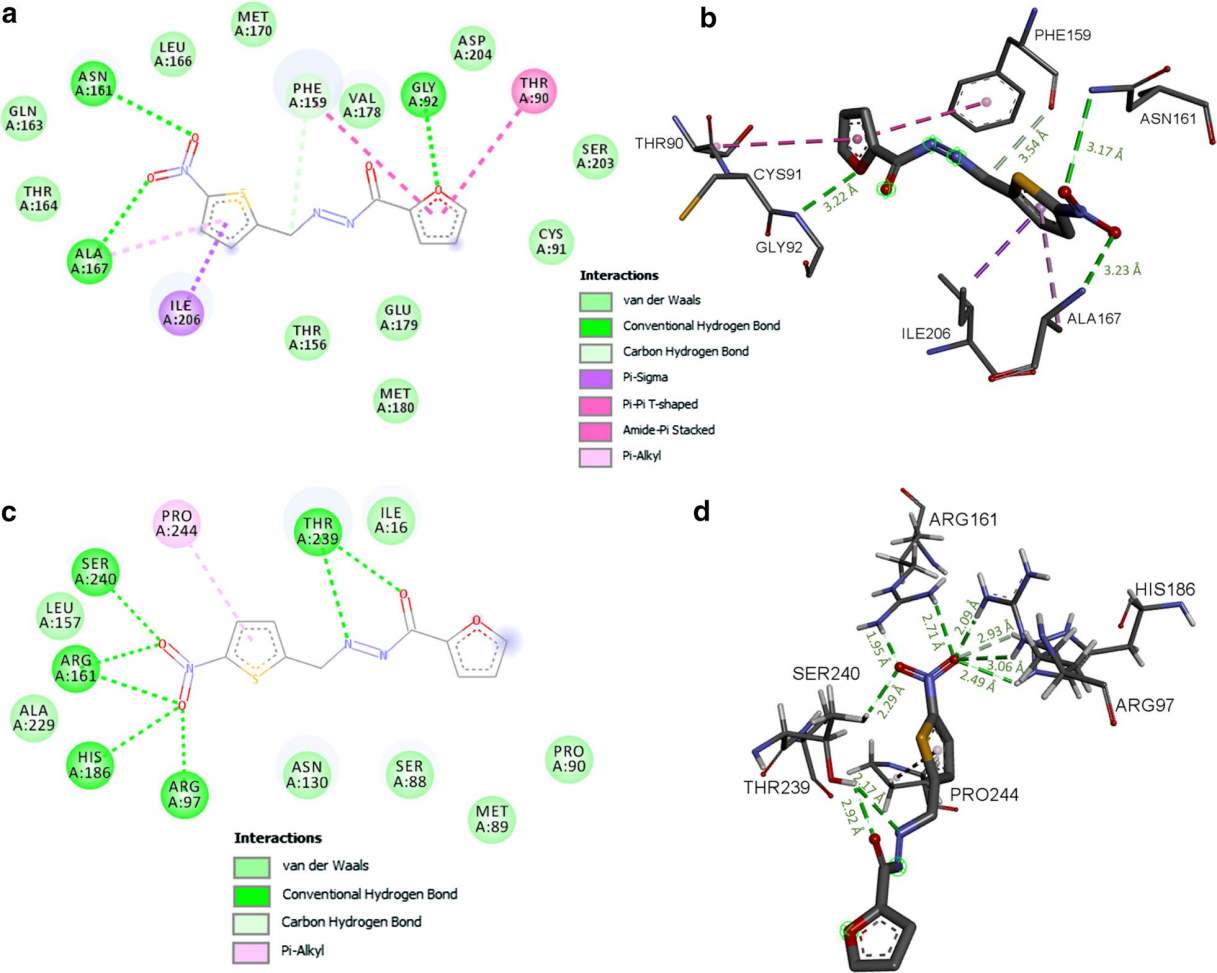
Representation of 2D projection and predicted binding mode of PFUR 4a with the *T. vaginalis* enzymes TvPNP (**a**, **b**) and TvLDH (**c**, **d**). The distance (Å) of the hydrogen bonds between specific residues and PFUR 4a is shown in green

We noticed that PFUR 4a interacts *in silico* with TvPNP with binding free energy (ΔG_bind_) of − 6.4 kcal/mol and four residues may be involved in hydrogen bonds with PFUR 4a (Fig. 5a, b, Table 2). As shown in Fig. 5c and d, PFUR 4a could possibly form eight conventional hydrogen bonds and one carbon-hydrogen bond (SER240: 2.29 Å) with active site residues of the enzyme TvLDH, alongside other non-covalent interaction that yield a docking score of − 5.8 kcal/mol (Table 2). We also performed docking studies of PFUR 4a with enzymes TvTrxR and TvMGL; however, no favorable binding mode was found (data not shown).

**Table 2:** Binding energies and molecular interactions predicted *in silico* between PFUR 4a, PFUR 4b, and MTZ, and enzymes important to *T. vaginalis* survival

Compound	Target enzyme	Binding energy (kcal/mol)	No. of hydrogen bonds	Hydrogen bond residues	Hydrogen bond length (Å)	Non-covalent interactions
PFUR 4a	TvPNP	− 6.4	4	GLY92, PHE159, ANS161, ALA167	3.22, 3.54, 3.17, 3.23	THR90, CYS91, THR156, PHE159, GLN163, THR164, LEU166, MET170, VAL178, GLU179, MET180, SER203, ASP204
PFUR 4a	TvLDH	− 5.8	8	ARG97, ARG161, HIS186, THR239	2.09, 3.06, 1.95, 2.71, 2.49, 2.93, 2.17, 2.92	ILE16, SER88, MET89, PRO90, ASN130, LEU157, ALA229
PFUR 4b	TvPNP	− 6.8	4	GLY20, ARG87, GLY89, THR90	3.02, 2.80, 3.57, 3.02	CYS19, ASP21, HIS62, VAL88, GLY89, CYS91, GLY92, THR156, PHE159, GLU179, ILE206
PFUR 4b	TvLDH	− 5.9	7	ARG97, ARG161, HIS186, SER240	2.12, 3.02, 2.24, 2.83, 2.54, 3.09, 2.09	GLY14, GLN15, ILE16, SER88, LEU91, ASN130, LEU157, ALA229, TRP230
PFUR 4b	TvTrxR	− 6.0	6	GLN82, LYS167, SER172, ALA173	3.35, 2.98, 3.19, 3.38, 2.87, 3.36	TYR85, THR86, LYS154, ALA155, TYR157, ASN168, ASP178
PFUR 4b	TvMGL	− 5.7	3	TYR111, ANS158, LYS209	3.04, 2.80, 3.27	SER85, GLY86, MET87, ILE90, ASP184, THR186, SER206, THR208, VAL218
MTZ	TvPNP	− 5.0	3	ASN161, GLN163, ALA167	2.89, 3.07, 2.98	PHE154, THR156, LEU158, PHE159, THR164, LEU166, MET170, VAL178, ILE206
MTZ	TvLDH	− 5.2	5	LEU157, ARG161, HIS186, SER240	2.08, 2.49, 2.30, 2.30, 2.34	ILE16, ILE128, ASN130, LEU154, ALA229, THR239, PRO244
MTZ	TvMGL	− 4.2	4	SER206, LYS209	2.99, 3.06, 2.98, 3.27	SER85, GLY86, MET87, TYR111, THR208, VAL218, VAL337, SER338

*Abbreviations*: MTZ, metronidazole; TvPNP, *T. vaginalis* purine nucleoside phosphorylase; TvLDH, *T. vaginalis* lactate dehydrogenase; TvTrxR, *T. vaginalis* thioredoxin reductase; TvMGL, *T. vaginalis* methionine gamma-lyase

**Fig. 6:**
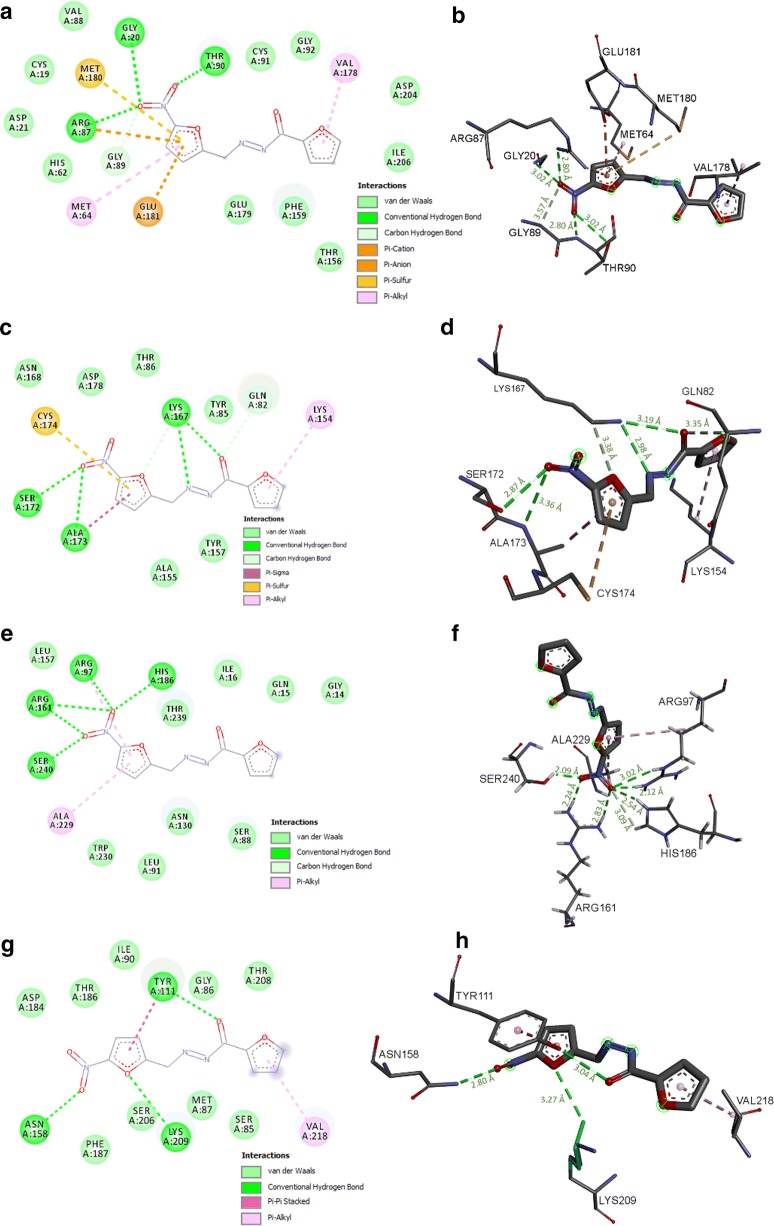
Representation of 2D projection and predicted binding mode of PFUR 4b with the *T. vaginalis* enzymes TvPNP (**a**, **b**), TvTrxR (**c**, **d**), TvLDH (**e**, **f**) and TvMGL (**g**, **h**). The distance (Å) of the hydrogen bonds between specific residues and PFUR 4b is shown in green

In the case of PFUR 4b, Fig. 6a and b show that it may form four conventional hydrogen bonds and one carbon-hydrogen bond (GLY89) with the active site residues of the enzyme TvPNP, alongside other non-covalent interactions that yield a docking score of − 6.8 kcal/mol (Table 2). PFUR 4b has a significant binding score of − 6.0 kcal/mol with TvTrxR as shown in Fig. 6c and d and it could be involved in six hydrogen bonds that might help in maintaining the binding mode (Table 2).

It is worth mentioning that PFUR 4b might potentially form seven hydrogen bonds with residues in the active site of TvLDH (Fig. 6e, f, and Table 2). This binding mode is complemented with hydrophobic interactions and Van der Waals forces, generating a ΔG_bind_ of − 5.9 kcal/mol (Table 2). Furthermore, a likely binding mode of PFUR 4b in the active site of TvMGL is shown in Fig. 6g and h (docking score of − 5.7 kcal/mol). Hydrogen bonds might be formed between PFUR 4b and three residues of TvMGL, and other chemical interactions may help in maintaining the binding mode of the compound (Table 2).

**Fig. 7:**
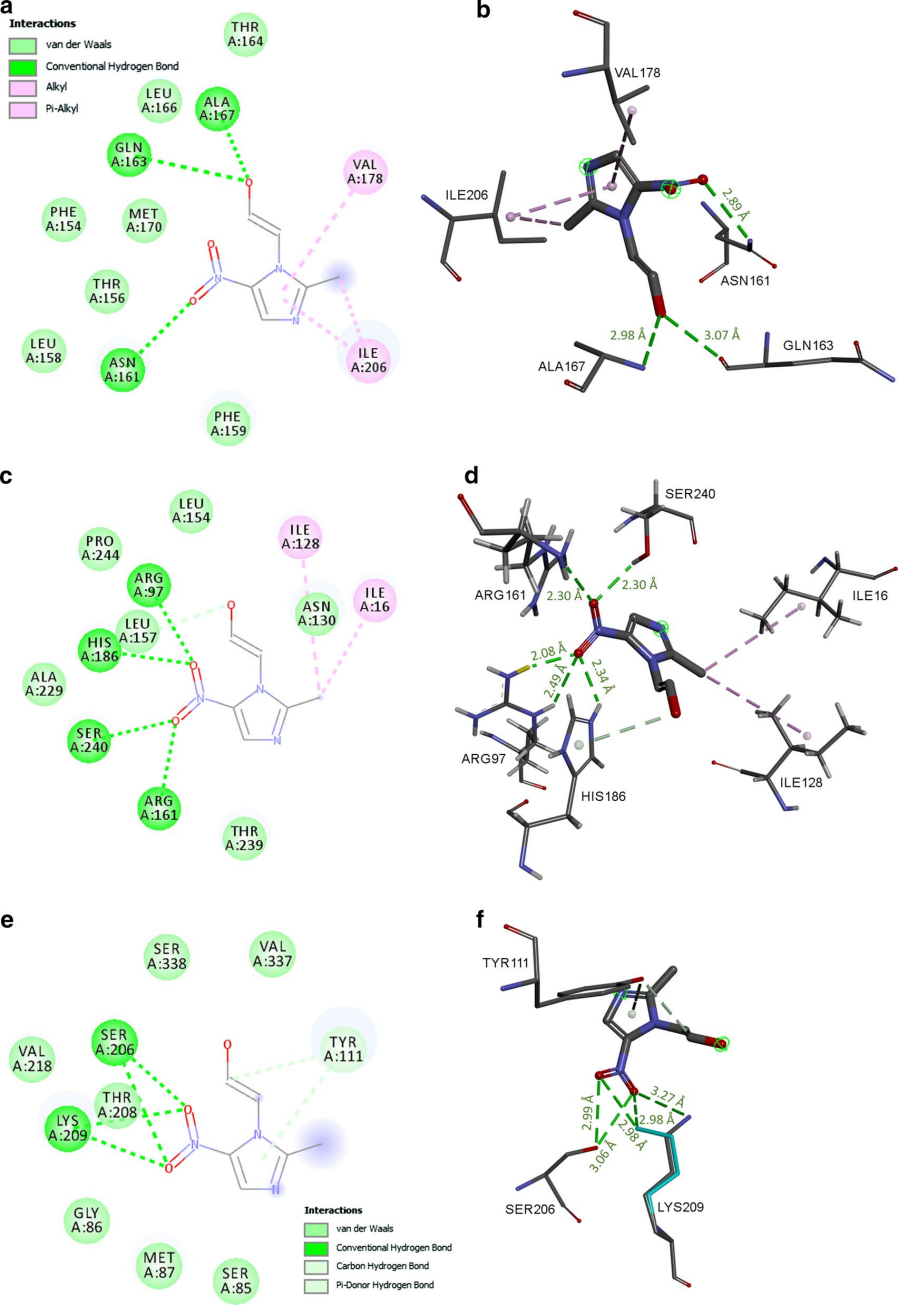
Representation of 2D projection and predicted binding mode of metronidazole (MTZ) with the *T. vaginalis* enzymes TvPNP (**a**, **b**), TvLDH (**c**, **d**) and TvMGL (**e**, **f**). The distance (Å) of the hydrogen bonds between specific residues and MTZ is shown in green

Considering that MTZ is the reference drug for *T. vaginalis* treatment, we also investigated its possible interaction with the abovementioned enzymes. *In silico* analysis showed MTZ possibly binds to the active site of TvPNP (ΔG_bind_ of − 5.0 kcal/mol; Fig. 7a, b), TvLDH (ΔG_bind_ of − 5.2 kcal/mol; Fig. 7c, d), and TvMGL (ΔG_bind_ of − 4.2 kcal/mol; Fig. 7e, f). The possible hydrogen bonds and other interactions of MTZ with the enzymes can be seen in Table 2.

### Cytotoxic assay

**Fig. 8:**
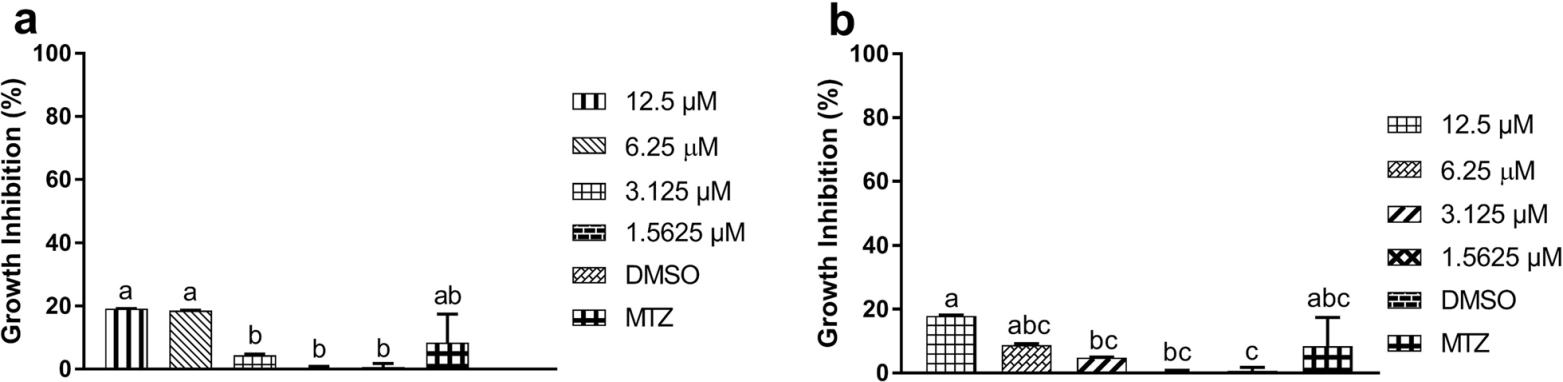
Cytotoxicity effect of PFUR 4a (**a**) and PFUR 4b (**b**) at 1.5625, 3.125, 6.25 and 12.5 µM on CHO-K1 cells through MTT assay after 24 h of exposure; 0.6% DMSO (vehicle for solubilization) and MTZ (metronidazole at 100 µM). Data are presented as the mean ± standard deviation of at least three independent experiments. Different letters show a significant difference between treatments at *P *< 0.05

The MTT assay showed that PFUR 4a induced 18.6% of growth inhibition at 6.25 µM and only 4.4% at 3.125 µM, none of the concentrations tested for this compound were significantly different from MTZ (ANOVA: *F*_(5, 12) _= 15.98, *P* < 0.0001), which caused 8.5% of inhibition (Fig. 8a). For PFUR 4b, the results demonstrated 8.8% of growth inhibition at 6.25 µM and 4.9% inhibition at 3.125 µM, once again none of the concentrations tested were significantly different from MTZ (ANOVA: *F*_(5, 12) _= 9.427, *P*= 0.0008) (Fig. 8b). As expected, 0.6% DMSO did not alter cell growth and 1.5625 µM concentrations of both compounds also had no effect on cells. The CC_50_ for both compounds was higher than the highest concentration tested (12.5 µM) (Table 1) because less than 50% of cell growth was inhibited at this concentration (Fig. 8a, b).

## Discussion

Trichomoniasis has a significant impact on public health due to its high incidence and prevalence worldwide [1]. The infection is associated with HIV acquisition and transmission, a higher risk of preterm delivery, and a higher prevalence of HSV-1 and HSV-2 [37–40], aggravating its effect on people’s health. In addition, with an increase in treatment failures resulting in recurrent infections, there is an obvious need to develop new alternatives for treating trichomoniasis. Our data showed two furanyl *N*-acylhydrazone derivatives, PFUR4a and 4b, presented *in vitro* antiprotozoal activity against *T. vaginalis* (Fig. 2) in promising concentrations and with a considerably good selectivity towards the parasite. Our *in silico* and *in vitro* analyses indicated that these two compounds are promising treatment alternatives, which may act slightly differently from the reference drug, and should be further explored.

In this context, we highlight that both PFUR 4a and 4b, which demonstrated a high trichomonacidal activity, presented a MIC value of 6.25 µM (Fig. 3a, b) and IC_50_ of 1.69 µM and 1.98 µM, respectively (Table 1). Moreover, a kinetic growth analysis showed that PFUR 4a and 4b, both at 6.25 µM (MIC), were able to fully reduce *T. vaginalis* viability to a level that was not significantly different from MTZ after a 24 h treatment (Fig. 3c, d). Similar kinetic growth inhibition profiles have been demonstrated in previous studies [28, 36]. The furanyl *N*-acylhydrazone derivatives tested in this study were previously investigated for their antitubercular potential and PFUR 4b exhibited moderate activity against *Mycobacterium tuberculosis* with a MIC of 100.3 µM [26]. When compared, these results suggest that these compounds might be more active/selective against protozoan species.

Other *N-*acylhydrazone derivatives have been reported as antiparasitic agents against *Trypanosoma cruzi in vitro* and *in vivo* in promising concentrations and also showed low cytotoxicity against mammalian cells in both cases [41], consequently demonstrating a great level of selectivity towards protozoan species. However, to the best of authors’ knowledge there are no prior reports of compounds containing the *N-*acylhydrazone moiety being active against *T. vaginalis*, or a species of the genus *Trichomonas*. As for the furan moiety, previous studies have shown that compounds containing this pharmacophoric group are active against *T. vaginalis* and *Giardia lamblia* [42, 43]. These studies substantiate the antiprotozoan potential of furanyl *N-*acylhydrazone derivatives demonstrated in our results.

The compounds PFUR 4a and 4b are quite similar in structure, the former having a 5-nitrofuran-2-yl radical and the latter a 5-nitrothiopen-2-yl radical, and both compounds are distinguished from the other 10 compounds by having this nitro (NO_2_) substituent in their structure. Similarly, MTZ also has a nitro group in its structure and this group plays an essential role in the production of toxic intermediates after its reduction and MTZ activation [44]. Considering these similarities, it has been speculated that nitro groups of PFUR 4a and 4b might play a role in altering the redox equilibrium in hydrogenosomes leading to the trichomonacidal activity reported here. Similarly, a previous study on *N-*acylhydrazones containing 4-hydroxy-3-methoxyphenyl showed antiparasitic activity against *Plasmodium falciparum* demonstrating that the addition of a nitro group significantly increased the compound’s activity, indicating that nitro groups could either induce or enhance antiparasitic activity against protozoan species [19]. In addition, the structural resemblance of compounds PFUR 4a and 4b could help elucidate the similar results observed in the *in vitro* antiparasitic and biochemical assays performed here.

Moreover, the TBARS assay demonstrated that *T. vaginalis* trophozoites exposed to PFUR 4a and 4b for 24 h did not present a level of lipid peroxidation significantly higher than the one observed for the control group (Fig. 4). Considering that lipid peroxidation is a chain of reactions mediated by free radicals that results in an oxidative deterioration of polyunsaturated lipids, which are abundant in components of biological membranes [45], these results indicate that membrane damage is not a significant factor to be considered as a mechanism by which these compounds induce *T. vaginalis* death.

Docking studies were performed to gain insight into the binding mode of compounds, PFUR 4a and PFUR 4b, with enzymes required for *T. vaginalis* survival. PFUR 4a presented a satisfactory binding mode with TvPNP and TvLDH, while PFUR 4b interacted *in silico* with TvPNP, TvTrxR, TvLDH and TvMGL (Figs. 5, 6 and Table 2). The results indicate that these enzymes could be somehow involved in these compounds’ action against *T. vaginalis*.

*Trichomonas vaginalis* PNP is a well-known potential target for chemotherapy [46, 47] and both compounds interact *in silico* with key residues in the active site of the enzyme, which are relevant for the inhibition of its activity, indicating they may act as TvPNP inhibitors. Based on the metabolic deficiency of *T. vaginalis* towards the lack of *de novo* synthesis of purine nucleosides and the requirement of purine salvage to maintain their purine nucleotide pools in order to survive [16], modulation of this pathway may be involved in the mechanism of action of PFUR 4a and PFUR 4b against *T. vaginalis*, highlighting the significance of this predicted interaction. Furthermore, based on the significance of TvLDH for parasite survival and *in silico* interactions described, impairment of energy metabolism could also explain the reduction in viability of the flagellated parasitic protozoan *T. vaginalis* by PFUR 4a and PFUR 4b.

Likewise, *in silico* interaction of PFUR 4b with key residues in the active site of TvTrxR and TvMGL could indicate a possible modulation of both enzymes, probably through their inhibition, by this compound. Considering the exclusivity of *in silico* interaction of PFUR 4b with TvTrxR and TvMGL when compared to PFUR 4a, we may hypothesize that modulation of antioxidant defense (through TvTrxR) and the content of sulfur-containing amino acids (*via* TvMGL) could account for the faster onset of action of PFUR 4b when compared to PFUR 4b (Fig. 3c, d). It is worth mentioning that TvMGL is a potential drug target for anti-*T*. *vaginalis* chemotherapy since it is not found in mammals, and the structure of TvTrxR is very different from human TrxR [18], highlighting the biological application of PFUR 4b. Nonetheless, more studies must be conducted to confirm this hypothesis and improve our understanding of the mechanism of action for PFUR 4a and PFUR 4b.

In order to establish a comparison between the putative effects of PFUR 4a and 4b and the known activity of MTZ, we also analyzed the possible binding mode of MTZ with the enzymes investigated here. Interestingly, the *in silico* interactions of TvPNP, TvLDH, and TvMGL were stronger with PFUR 4a and 4b than with MTZ (Fig. 7 and Table 2). In *T. vaginalis*, among the actions described [11, 44, 48], MTZ is said to disrupt the DNA helical structure inhibiting the synthesis of proteins that result in cell death [44, 48]; therefore, inhibiting the enzymes investigated here may not be required for the action of MTZ. This indicates that the mechanism of action of PFUR 4a and 4b might be somewhat different from the mechanism of action of MTZ, highlighting that PFUR 4a and 4b may act *via* modulation of energy metabolism, antioxidant defenses, and the content of sulfur-containing amino acids. Nonetheless, more studies are needed to support this hypothesis.

The cytotoxicity assay performed in CHO-K1 cells showed that at their MIC (6.25 µM), both compounds exhibited no cytotoxic effect on non-tumor/normal cells (Fig. 8a, b) according to the standards set by the International Organization for Standardization (ISO) at ISO 10993-5:2009, which states that a decrease of cell viability by more than 30% is considered a cytotoxic effect [49, 50]. Therefore, since PFUR 4a and PFUR 4b reduced viability by 18.6% and 8.8% at their MIC, respectively, this inhibition of growth does not represent a cytotoxic effect. In addition, levels of cell growth inhibition are significantly the same as the one found for MTZ, demonstrating that these compounds do not affect cells more than the commercial drugs used for trichomoniasis. Lastly, both compounds showed a promising SI (Table 1), which can be much better than currently presented considering that the higher concentration tested (12.5 µM) inhibited less than 20% of the mammalian cells. Altogether, these results emphasize the potential application of these molecules.

Finally, the need for new and effective molecules for treating trichomoniasis is emerging exponentially and all data gathered here show that furanyl *N-*acylhydrazone derivatives, PFUR 4a and 4b, have a highly significant antiparasitic activity against *T. vaginalis* with a great level of selectivity for the parasite over non-tumor/normal cells and are, therefore, promising molecules for studies on new trichomonacidal agents.

## Conclusions

Our study demonstrates that furanyl *N*-acylhydrazone derivatives PFUR 4a and PFUR 4b have a potent anti-*T. vaginalis* activity *in vitro*, which is statistically similar to the reference drug. At low concentrations, these compounds show good and promising selectivity towards *T. vaginalis*. Moreover, molecular docking analyses point to the modulation of enzymes TvPNP, TvLDH, TvTrxR, and TvMGL by these compounds. In conclusion, our data characterized PFUR 4a and PFUR 4b as promising trichomonacidal agents and support further studies not only with these compounds but also other *N*-acylhydrazone derivatives, as alternatives for treating trichomoniasis.

## Data Availability

Data supporting the conclusions of this article are provided within the article. The datasets used and/or analyzed during the present study are available from the corresponding author upon reasonable request.

## Abbreviations

PFUR: Furanyl *N*-acylhydrazone derivatives
STI: Sexually transmitted infection
ATCC: American type culture collection
MIC: Minimum inhibitory concentration
IC_50_: 50% inhibitory concentration
CC_50_: 50% cell inhibitory concentration
WHO: World Health Organization
TvPNP: *T. vaginalis* purine nucleoside phosphorylase
TvLDH: *T. vaginalis* lactate dehydrogenase
TvMGL: *T. vaginalis* methionine gamma-lyase
TvTrxR: *T. vaginalis* thioredoxin reductase
MTZ: Metronidazole
DMSO: Dimethylsulfoxide
TBARS: Thiobarbituric acid reactive substances
MDA: Malondialdehyde
TBA: Thiobarbituric acid
CHO-K1: Chinese hamster ovary cells
MTT: 3-(4,5-dimethythiazol-2-yl)–2,5-diphenyl tetrazolium bromide
SI: Selective index
